# Immobilization of Biomass Materials for Removal of Refractory Organic Pollutants from Wastewater

**DOI:** 10.3390/ijerph192113830

**Published:** 2022-10-24

**Authors:** Danxia Liu, Xiaolong Yang, Lin Zhang, Yiyan Tang, Huijun He, Meina Liang, Zhihong Tu, Hongxiang Zhu

**Affiliations:** 1 Guangxi Key Laboratory of Environmental Pollution Control Theory and Technology, College of Environmental Science and Engineering, Guilin University of Technology, Guilin 541004, China; 2 Collaborative Innovation Center for Water Pollution Control and Water Safety in Karst Area, Guilin University of Technology, Guilin 541004, China; 3 Guangxi Key Laboratory of Environmental Pollution Control Theory and Technology for Science and Education Combined with Science and Technology Innovation Base, Guilin University of Technology, Guilin 541004, China; 4 CAS Key Laboratory of Mineralogy and Metallogeny, Guangzhou Institute of Geochemistry, Chinese Academy of Sciences, Guangzhou 510640, China; 5 Guangxi Modern Industry College of Ecology and Environmental Protection, Guilin 541006, China

**Keywords:** immobilization, microorganisms, enzyme, organic pollutants, bioreactor

## Abstract

In the field of environmental science and engineering, microorganisms, enzymes and algae are promising biomass materials that can effectively degrade pollutants. However, problems such as poor environmental adaptability, recycling difficulties, and secondary pollution exist in the practical application of non-immobilized biomass materials. Biomass immobilization is a novel environmental remediation technology that can effectively solve these problems. Compared with non-immobilized biomass, immobilized biomass materials have the advantages of reusability and stability in terms of pH, temperature, handling, and storage. Many researchers have studied immobilization technology (i.e., methods, carriers, and biomass types) and its applications for removing refractory organic pollutants. Based on this, this paper reviews biomass immobilization technology, outlines the mechanisms and factors affecting the removal of refractory organic pollutants, and introduces the application of immobilized biomass materials as fillers for reactors in water purification. This review provides some practical references for the preparation and application of immobilized biomass materials and promotes further research and development to expand the application range of this material for water purification.

## 1. Introduction

In recent years, environmental pollution caused by human activities has become an increasingly serious problem, posing a serious threat to human health and the environment [1,2]. Various types of organic pollutants (e.g., dyes, phenols, pesticides, drugs, and hormones) discharged in human production and life have seriously polluted water bodies, and many of them can accumulate in organisms, leading to adverse effects on growth, development, and metabolism [3,4]. In addition, refractory organic pollutants are highly stable and biotoxic, and most have been shown to cause diseases such as cancer, cardiovascular diseases, and reproductive disorders [5,6]. Therefore, it is critical to remove these organic pollutants before they are discharged into an aqueous environment. Commonly used wastewater treatment methods include physical and chemical methods (such as adsorption, electrocatalysis, advanced oxidation, and membrane filtration) and their combined techniques [7]. Although these methods can achieve high removal rates, there are still some limitations to their practical applications. The disadvantages associated with these conventional methods include complete removal of pollutants, time consumption, sludge generation, and high energy requirements [8]. Therefore, it is necessary to find easy-to-operate and cost-effective alternative solutions.

Bioremediation is an effective technique for treating pollutants in water and soil environments. It has the advantages of low cost, no secondary pollution, high efficiency, and eco-friendly recycling resources [9]. Currently, the desired bioremediation materials can be obtained from various sources, such as the plants *Acalypha indica* [10], *Saccharomyces* [11], *Aspergillus* [12], *white-rot fungi* [13], and algae [14,15], which have been widely used for the removal of organic pollutants from wastewater. In addition, it has been reported that peroxidases [16] and laccases [17,18] among biological enzymes also degrade organic pollutants; however, existing studies have shown that free microorganisms/enzymes have low stability, short service lives, and are not easy to separate and recover, which limits their further industrial application [19].

Immobilization is a technique that confines biomass materials (microorganisms or enzymes) to a certain spatial extent by physical or chemical means so that they cannot move freely and maintain their activity, facilitating their separation and recovery [20]. Immobilized biomass materials are significantly enhanced in terms of storage, operational stability, and reusability compared with free microorganisms/enzymes [21]. Immobilization techniques have been widely used in environmental management for the treatment of pollutants. Among them, biofilm method is a membrane immobilization method that uses immobilization of active bacteria to form biofilms for efficient removal of pollutants, and has been widely used in the field of water pollution treatment. Studies have shown that immobilization techniques can increase the biodegradation rate, especially in harsh environments [22]. For example, immobilized laccase can completely remove 100 mg/L of bisphenol A (BPA) in 4.0 h and has better thermal stability and reusability than free laccase does [23]. Immobilized microalgae (such as chlorella, red alga, marine diatom etc.) are highly effective in removing nitrogen and phosphorus from water [24]. Saccharomyces is a commonly used microorganism for immobilization, and studies have shown that immobilized *Saccharomyces cerevisiae* (*S. cerevisiae*) can remove up to 86.23% of antibiotics [25]. In addition, immobilization technology has applications in biosensors [26,27], medical imaging, biocatalysis [28], cancer therapy [29], and cell delivery [30].

In recent years, many studies have been conducted on immobilization techniques and immobilized biomass materials for the treatment of refractory organic wastewater [31,32], but very few reviews of this research have been published. Therefore, based on previous studies and achievements of the authors in this field, this paper reviews the characteristics of immobilization methods, carriers, and biomass materials in the immobilization process, analyzes the effects of different operational factors in the preparation of immobilized biomasses, and presents applications of immobilized biomass materials in the treatment of refractory organic wastewater and its degradation mechanisms. Finally, challenges and possible future research directions in this field are briefly discussed.

## 2. Overview of Immobilization Technology

Biomass materials (such as microorganisms, enzymes) have been widely used for environmental protection because of its ease of production, greenness, and high environmental economic efficiency [33]. However, these biomass materials (such as saccharomycetes) are often difficult to recycle and have poor practicability. Many biomass materials have high requirements for the external environment, which affects their wide-scale use [34]. Immobilization technology is an effective way to solve these problems. Commonly used immobilization techniques include adsorption, covalent binding, entrapment, and cross-linking [35,36]. In this section, immobilization methods, carrier materials, and biomass types used in immobilization techniques are discussed.

### 2.1. Immobilization Methods

Adsorption and entrapment are physical immobilization methods, cross-linking and covalent binding are chemical immobilization methods [8], and the biofilm method is the physicochemical immobilization method, with entrapment being one of the most commonly used methods. Each immobilization method has specific operating conditions; therefore, the selection of an appropriate immobilization technique is a key factor for success [37], and inappropriate immobilization can lead to structural changes, blockage of the active site, and blocked mass transfer, which in turn leads to a loss of material activity.

#### 2.1.1. Adsorption

**Figure 1:**
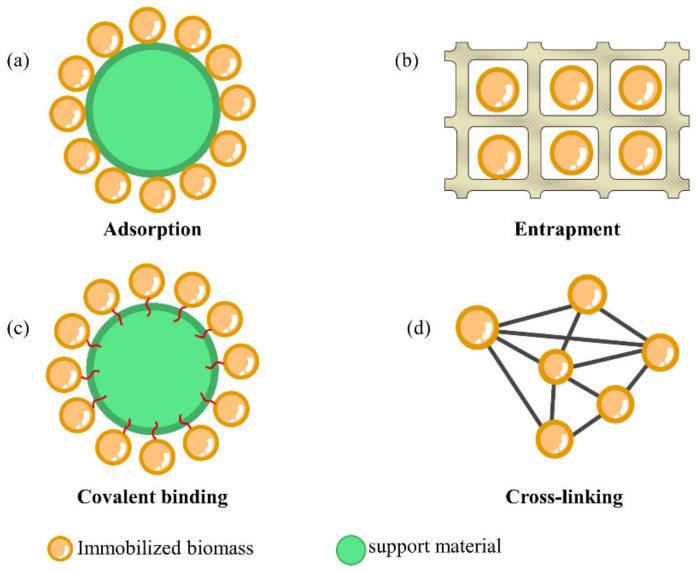
Illustrative scheme showing immobilization methods: (**a**) adsorption, (**b**) entrapment, (**c**) covalent binding, (**d**) cross-linking.

Compared to other immobilization methods, the adsorption immobilization method is simple and low cost. It mainly immobilizes biomass materials on the surface or inside the carrier material through interactions such as hydrogen bonding, hydrophobic interactions, electron affinity, and van der Waals forces [38] without changing the natural structure of the biomass, as shown in Figure 1a. Commonly used adsorption carrier materials include activated carbon, kaolin, porous glass, bentonite, and the corresponding modified materials.

Yan et al. [39] utilized graphene oxide (GO) to immobilize *Enterococcus avium* strain BY7 sulfate-reducing bacteria and found that the addition of GO accelerated the growth rate of the strain, with a maximum growth rate of about 0.27/h and a maximum doubling time of about 2.5 h at GO addition of 12.0 mL/L; the growth rate of the strain was three times higher than it was without the addition of GO. Wen et al. [40] utilized acid-base modified bentonite (BDMMs) to immobilize laccase by adsorption and demonstrated that the specific surface area of the modified bentonite reached 244.62 m^2^/g, which is 74 times higher than that of the original bentonite, while laccase activity reached 800 U/g at pH 4–5, which led to a significant improvement in the thermal and operational stability of laccase.

Adsorption immobilization has certain advantages in terms of preparation, operating conditions, and reuse, but it also has some disadvantages, such as a weak affinity between microbial cells/enzymes and carriers, which leads to easy shedding of the loaded biomass, thus decreasing the immobilization efficiency and reducing its effect on pollutant removal. Therefore, it is necessary to develop carriers with strong adsorption capacity to improve the immobilization effect when this technology is employed.

#### 2.1.2. Entrapment

Entrapment is one of the most commonly used methods to prepare immobilized materials (Figure 1b); it involves immobilizing biomass materials in a carrier to form small particles, preventing leakage, and reducing the effect of adverse external environments on the biomass materials. Entrapment methods can be divided into gel entrapment and semi-permeable membrane/capsule entrapment. Entrapment materials include natural gels (e.g., alginate, gelatin, chitosan) and synthetic polymers (polyvinyl alcohol, polyacrylamide), of which alginate is the most commonly used natural polymer carrier material because of its porosity and high biocompatibility [41]. Wu et al. [42] utilized sodium alginate (SA) and polyvinyl alcohol (PVA)–encapsulated magnetic-Fe_3_O_4_-immobilized *S. cerevisiae* to remove atrazine; they found that the removal of atrazine by the materials prepared using this technique could reach 92%. In addition, the encapsulation method can be used for the preservation of strains, which can conserve the activity of cells for a long time under ultra-low-temperature storage conditions [43].

Entrapment can protect biomass materials from the external environment and prevent leakage of microorganisms and enzymes [33]. However, because the entrapment method limits the mass transfer efficiency between the biomass materials and the external environment, which may affect the activity of the biomass, it is most suitable for reaction systems with small molecular substrates.

#### 2.1.3. Covalent Binding

**Figure 2:**
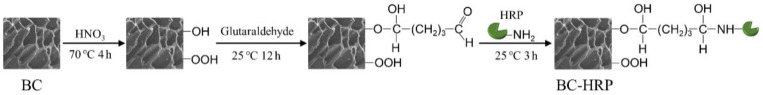
Procedure of covalent immobilization of HRP onto biochar.

Covalent binding (Figure 1c) is based on the formation of covalent bonds between chemical groups on the surface of the carrier and the nucleophilic groups on the biomass material [44], which is particularly suitable for immobilizing biomasses to enhance stability. For example, Petronijević et al. [45] prepared biochar (BC)-immobilized horseradish peroxidase (HRP) for the biodegradation of phenol (Figure 2), first oxidizing the BC using a nitric acid solution to introduce the hydroxyl group and binding it to the aldehyde group at one end of the molecular chain of glutaraldehyde (cross-linked). Then, the amino group of the enzyme was combined with the free aldehyde group at the other end of glutaraldehyde via a strong covalent bond to complete the fixation of HRP. This study demonstrated that the prepared BC-HRP had higher storage and thermal stability than the non-immobilizing HRP.

Simon-Herrero et al. [46] immobilized laccase on modified polyimide aerogels via covalent bonding. The study showed that the activity of the immobilized enzyme was consistently higher than that of the free enzyme, whereas the effective binding efficiency of covalent immobilization was 3.6 times higher than that of absorbed immobilization. In addition, some studies have found that the use of nanomaterials with large specific areas as additives could further enhance the biomass-loading capacity of carriers in the covalent bonding method. For example, Skoronski et al. [47] immobilized laccase using graphene nanomaterials modified with NH_2_ groups and used glutaraldehyde to covalently immobilize the graphene and enzymes. The results showed that the immobilized laccase had a much higher range of adaptation to the environmental pH and temperature than the free enzyme did. Meanwhile, the covalently immobilized laccase still had good operational stability and enzymatic activity after six repeated uses.

Because there is a chemical reaction process in covalent bonding, when using covalent binding to immobilize microorganisms, the cells are exposed to chemicals that can easily damage the cells and reduce the metabolic activity of the microorganisms. Therefore, covalent bonding is more suitable for immobilizing inactive cells [8]. Overall, this immobilization method exhibits a higher affinity between the carrier and the biomass materials, as well as better stability, but the method is more complicated to operate and has harsh conditions [19], making it less suitable for industrialization. Covalent bonding can lead to changes in the spatial structure of the biomass materials, which may alter its original biological properties and functions and cause biological activity loss [48].

#### 2.1.4. Cross-Linking

Compared to covalent bonding, the cross-linking method (Figure 1d) is more widely applicable and has strong bonding, high stability, and simple operation. Cross-linking agents play an important role in cross-linking immobilization. Commonly used cross-linking agents include glutaraldehyde, diacetamide, hexanediamine, maleic anhydride, and isocyanate derivatives.

Dzionek et al. [49] used xanthan gum to cross-link immobilized microbial cells *Bacillus thuringiensis* B1 and hardened them using polydopamine, which showed that the material could completely remove naproxen within 14 days, while no by-products were produced during degradation. Lee et al. [50] utilized inorganic calcium carbonate to cross-link and immobilize carboxyl esterase. They demonstrated that the cross-linked enzyme exhibited higher activity than the free enzyme did and maintained 60% of the enzyme activity after 10 reuse cycles, indicating that cross-linked immobilization enhanced the stability of the enzyme. Guo et al. [51] prepared an immobilized material, MDCIL, by immobilizing Rhizopus lipase on magnetic nanoparticles using dialdehyde cellulose (DAC) as a cross-linking agent. In this study, it was found that the immobilization yield and recovery of lipase were 60% and 89%, respectively, under optimal conditions. In addition, MDCIL had better thermal and storage stability than the free enzyme did, which is mainly due to the increase in the secondary structural rigidity of MDCIL due to immobilization.

Unlike adsorption, cross-linking is an irreversible process, and cross-linking agents used are usually cytotoxic, leading to the loss of microbial activity and making cross-linking more successful in the immobilization of inactive microbial cells or enzymes [33].

In practice, multiple methods are used together to address the shortcomings of a particular technique, such as adsorption–covalent [52,53] and covalent–cross linking [54]. Combining multiple immobilization methods can effectively improve the stability of microorganisms and enzymes as well as their adaptability to the environment. 

#### 2.1.5. Biofilm Method

The biofilm method is used to immobilize microbial community groups on the carrier through surface adsorption and inner fixation, gradually forming biofilm. The growth of biofilm on the carrier accumulates and sheds, so as to achieve continuous degradation of pollutants [55]. 

In the application, the selection of biological carriers is the key factor affecting the immobilization. The physical properties, chemical stability, particle size, and porosity of the carriers have an important influence on the biofilm method, and the carriers usually used to immobilize biofilms are inorganic materials such as quartz sand, activated carbon, ceramic granules, and some organic materials such as polyethylene, polypropylene, and PVC. Wang et al. [56] used 20 mesh stainless steel sieve as a carrier to immobilize mixed microorganism as biofilm, and it was found that phthalates and bisphenols were easily adsorbed onto the biofilm with the action of microorganisms. In addition to the influence of the carrier, microbial activity is also the key factor in the application of biofilm. Derakhshan et al. [57] utilized biofilm to remove atrazine and showed that under optimal conditions, 60% of the atrazine was removed, but when increasing the concentration of atrazine, the microorganism activity in the biofilm was inhibited and the removal of atrazine was decreased by 12%. 

The core of the biofilm method is the carrier, which plays the role of fixing microorganisms and directly affects the operating effect of the biofilm reactor. Therefore, it is necessary to choose the carrier materials with good effect. 

### 2.2. Immobilization Carriers

In general, the mechanical strength and mass transfer properties of immobilized biomasses are affected by the immobilization method as well as the carrier material. Therefore, the selection of a suitable immobilized carrier to meet the high affinity for biomass materials (microorganisms/enzymes) and the high contaminant removal capacity is also a key factor [58,59]. Simultaneously, it should be noted that carriers should be environmentally friendly, chemically and mechanically stable, and have excellent biocompatibility [60]. Various types of carriers are available at present, which can be broadly classified as traditional carriers (such as activated carbon, clay, zeolite, etc.) and novel carriers (such as nanomaterials, magnetic materials, and mesoporous materials). Table 1 shows the changes in enzyme activity, thermal stability, storage stability, and operational stability before and after immobilization.

#### 2.2.1. Traditional Carriers

Traditional carriers include inorganic, organic and composite materials such as activated carbon (AC), clay, chitosan, agar, and silica. Mineral materials are common inorganic carriers with low cost and easy accessibility, excellent thermal and chemical stability, and a high number of adsorption sites [61]. Among them, carbon-based materials have great potential for practical applications owing to their high thermal and chemical stability [62]. It has been shown that the addition of BC during the immobilization process can reduce inter and ionic repulsion and enhance the binding ability between microorganisms and the carrier, thereby improving the efficiency of atrazine removal [63]. Chen et al. [64] improved the performance of immobilized microorganisms and enhanced the mechanical strength and mass transfer efficiency of immobilized beads by adding AC. The study found that the material increased the degradation rate of crude oil by 8%, indicating that the AC carrier provided a good platform for microbial degradation. In most cases, the use of inorganic materials for immobilization is prone to unstable fixation and loss of biomass; therefore, it is necessary to improve the effective immobilization of inorganic materials. By changing the surface groups, specific surface areas, or pore structures of the inorganic carriers, they can be used in combination with other polymer materials to obtain a carrier with better performance [65].

Organic carriers can be divided into natural polymeric materials (e.g., agar, gelatin, alginate, and chitosan) and synthetic polymeric materials (e.g., polyvinyl alcohol, polyacrylamide, and polyurethane sponges) [66]. Compared to synthetic polymers, natural polymeric materials have superior diffusion efficiency, better biocompatibility, and lower costs [67,68]. Researchers have found that the amount of SA significantly affects the apparent morphology and mass transfer rate of immobilized materials, which in turn affects the degradation of pollutants [69], indicating that the carrier material is an important aspect affecting the performance of immobilized materials. In addition, synthetic organic materials have higher mechanical strengths and more stable chemical properties than natural organic polymers do [33]. Su et al. [70] investigated the immobilization of *Pseudomonas* sp. H117 using modified polyvinyl alcohol (PVA) and found that the addition of PVA facilitated bacterial immobilization and biofilm formation with longer retention and higher microbial cells metabolic activity.

Organic materials are easily decomposed by the external environment, leading to immobilization failure in practical applications; some organic materials are toxic to biomasses, which limits their widespread use. These problems are also technical issues that need to be solved when organic carriers are applied.

**Table 1:** Performance parameters of various free and immobilized enzymes.

Enzyme and Carrier	Enzyme Activity	Thermal Stability	Storage Stability	Operation Stability	Ref.
Free Laccase	/	70 °C, 6 h, 15%	4 °C, 28 d, 40.2%	/	[71]
Laccase on Fe_3_O_4_@CS nanoparticles	114.2 U/mg	70 °C, 6 h, 35%	4 °C, 28 d, 75.2%	5cycles: 36%	
Free Chymotrypsin	/	60 °C, 3 h, 29.6%	4 °C, 20 d, 18.8%	/	[71]
Chymotrypsin on magnetic Chitin Nanofiber Composite	/	60 °C, 3 h, 70.7%	4 °C, 20 d, 84.9%	5cycles: 78.6%	
Free Porcine pancreatic lipase	/	60 °C, 26%	4 °C, 56 d, 20%	/	[72]
Porcine pancreatic lipase on 3D,GO/PVA/Fe_3_O_4_	/	60 °C, 64%	4 °C, 56 d, 71.1%	6cycles: 70.8%	
Free Laccase	85.9 U/g	40 °C, 55%	25 °C, 20 d, 4%	/	[46]
Laccase on polyimide aerogels	8.0 U/g	40 °C, 98%	25 °C, 20 d, 20%	7cycles: 22%	
Free Inulinase	33.8 U/mg	60 °C, 3 h, 33.8%	4 °C, 6 w, 44.3%	/	[73]
Inulinase on shallow porous microsphere carriers	24.7 U/mg	70 °C, 3 h, 69.2%	4 °C, 6 w, 71.4%	10cycles: 77.9%	

Note. d—days, U—Enzyme activity units, 1 U = 16.67 nkatal, w—weeks.

#### 2.2.2. Novel Carriers

Currently, magnetic materials, mesoporous materials, and metal–organic frameworks (MOFs) are considered novel carriers. Compared with traditional carriers, novel carriers have special structures and functions, such as magnetic and electrical conductivity; thus, they have unique advantages for immobilizing biomass.

In recent years, magnetic materials with high specific surface areas and high loading capacities have been considered as promising immobilization carriers that can be easily separated and recovered from the reaction system by applying an external magnetic field [74]. A new magnetic carrier, α-Fe_2_O_3_, was developed to immobilize *B. encimensis* and *B. badius*, and the results showed that the immobilized material achieved 90% removal of atrazine within 20 days; in addition, the microorganisms immobilized on the magnetic material were found to have better tolerance to temperature and pH compared to free microorganisms [75]. For example, Li et al. [76] used glutaraldehyde to immobilize horseradish peroxidase (HRP) and magnetic nanofibers (MNFs) to prepare immobilized material H-MNFs, which successfully achieved 85% removal of phenol and maintained 52% removal after five cycles. It has been demonstrated that some magnetic materials used as immobilization carriers also participate in the pollutant removal process to further improve the performance of immobilized materials [77]. Combining microorganisms/enzymes with magnetic materials is more helpful for the simple and effective recovery of immobilized materials, making the application of immobilization technology in industry more feasible. However, magnetic materials are generally made of metallic raw materials, most of which have certain toxic effects on biomass materials that when immobilized will be released into the environment and produce secondary pollution when applied; therefore, the development of low-toxicity or non-toxic magnetic materials is crucial. In addition, the cost of preparing magnetic particles is also a challenge for commercial applications.

**Figure 3:**
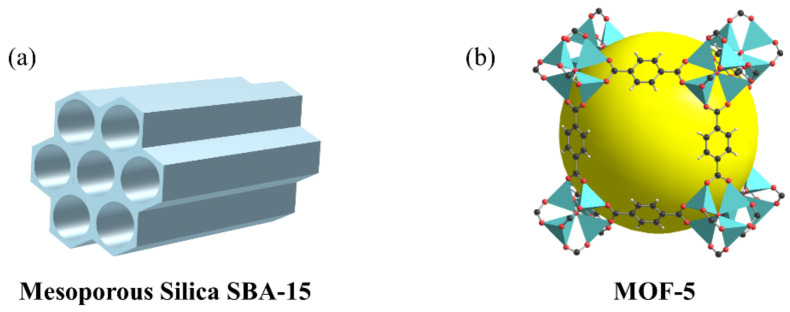
Structural diagram of typical novel carrier material: (**a**) mesoporous silica SBA-15, (**b**) MOF-5.

Mesoporous materials have a regular and ordered rigid pore structure, and their pore size and arrangement can be adjusted by changing the synthesis parameters, which can significantly increase the load of microorganisms/enzymes, making them effective immobilization carriers for many biomasses [78]. SBA-15 is a hexagonal mesoporous silica (Figure 3a) with a high specific surface area and pore size that is often used for enzyme immobilization. Kuo et al. [79] immobilized *Streptomyces griseus* HUT 6037 in three different mesoporous matrices: mesoporous silica film, mesocellular foam, and rod-like SBA-15. Their study demonstrated that all three immobilized materials exhibited more than 90% enzyme loading at optimal pH with efficient performance and reusability, indicating that the mesoporous materials improved the chemistry and stability of the enzyme. Parmegiani et al. [80] modified silica with tin (SnS23) to obtain SBA-15 with a pore size of 25 nm and used it as a carrier for immobilized lipase. The modified silica surface generated a Si-O-Sn-Cl chemical bond that reacted with the sulfhydryl group in the lipase molecule to form a strong covalent bond. The covalent bond between the enzyme and carrier can prevent desorption of the protein and enhance the stability of the immobilized lipase. The preparation process is demanding for obtaining the desired pore size; thus, carrier materials are generally expensive, which limits their application.

MOFs are emerging crystalline porous materials that are organic–inorganic hybrid materials composed of organic ligands and metal ions/clusters through covalent bonds [81]. More than 2000 MOFs have been reported, and MOF-5 (Figure 3b) is a common MOFs material first synthesized by Li et al. in 1999, which can have a specific surface area of up to 2900 m^2^/g [81]. The high porosity, open active sites, adjustable pore size, and mild synthesis conditions of MOFs make them excellent candidates for the immobilization of biomass materials, which have been extensively studied in recent years [82].

Researchers have used ZrCl_4_ and Fe-TCPP (where TCPP is tetrakis (4-carboxyphenyl) porphyrin) to prepare a multilayer porous material HP-PCN-224 (Fe) (where PCN stands for “porous coordinated network”) and applied it to the immobilization of natural enzymes, greatly improving the stability and loading capacity of immobilized enzymes [83]. Nowroozi-Nejad et al. [84] immobilized luciferase on MOF using benzaldehyde and found that the thermal stability and kinetic properties of the immobilized enzyme were greatly improved. However, the pore size of the MOFs carrier affects the loading capacity and mass transfer performance of the immobilized biomass, thereby affecting the interaction between biomass materials and carriers. Therefore, the design and regulation of the pore size and surface interface characteristics are important research directions.

For immobilized biomass, different carrier materials have different advantages, such as biocompatibility, mechanical stability, environmental friendliness, cost effectiveness, and feasibility of industrial applications [85]. Therefore, the structure of the carrier material, characteristics of the biomass, properties of the pollutants, and their removal conditions should be comprehensively considered when selecting the carrier. In practice, two or more materials can be combined by physical or chemical means to form a composite-carrier material to solve the defects existing in a single material and to optimize the performance of the immobilized carrier [86]. For example, considering the high biocompatibility and excellent stability of organic carriers, as well as the stability and chemical inertness of inorganic carriers, the combination of these two materials to obtain composite-carrier materials has been used to develop more efficient carriers. Girelli et al. [61] demonstrated that immobilizing laccase in silica–chitosan composites as carriers had higher thermal stability, and immobilized laccase maintained 40% of its enzyme activity after 200 days. Therefore, this is noteworthy for the study of stable performance, high biocompatibility, and affordable composite-carrier materials.

### 2.3. Biomass Materials Type

Immobilization techniques can be applied to different types of biomass, including microorganisms, enzymes, and whole cells, and they play an important role in environmental remediation, biotechnology, and biomedicine. In environmental remediation, the performance of immobilized materials is not only related to the carrier but also to the biomass materials and its suitability for pollutant removal [87]. For example, laccase and peroxidase are commonly used for phenolic compound removal and dye decolorization [88], and bacteria, fungi, and algae are widely used in water pollution, air control, and soil pollution treatment [89].

#### 2.3.1. Microorganisms

Microorganisms in environmental remediation can not only remove organic pollutants, heavy metals, and pathogens but can also remove odors, improve water transparency, and reduce chromaticity. However, free microbial cells are less stable, less adaptable and tolerant to the external environment, and difficult to recycle and reuse. Immobilization is an environmentally friendly and efficient technical means of solving these problems and has been valued by many researchers (Table 2).

Chen et al. [90] immobilized phosphate-solubilizing bacteria (PSB) on BC to remove Pb^2+^ from organic media. The study showed that the immobilization of BC significantly enhanced the removal of Pb^2+^ by PSB, and the addition of PSB also enhanced the release of phosphorus from the surface of BC to regulate environmental pH and improve the adsorption of Pb^2+^. Liu et al. [91] immobilized mixed microbial MO (mainly composed of *Pseudomonas* and *Delftia*) in chitosan and polyvinyl alcohol beads (MO/PVA-CS), which achieved a phenol degradation efficiency of 99.5% in 120 h, while the degradation rate of phenol by free MO under the same treatment conditions was only 21.1%, indicating that the activity of MO can be improved by immobilization. Wu et al. [42] prepared a novel bio-nanomaterial using magnetic Fe_3_O_4_ and SA-PVA-immobilized *S. cerevisiae* to remove atrazine. The study found that the presence of active *S. cerevisiae* greatly improved the removal of atrazine, with a maximum removal rate of 97.7%, which also indicated that the removal of atrazine by this novel material was mainly due to the biodegradation and metabolism of *S. cerevisiae*. Wang et al. [92] immobilized *Escherichia coli* on magnetic pellets for tanning wastewater treatment and found that the removal of Cr(III) from the water by the immobilized bacteria reached 91.3%, which was much higher than the adsorption efficiency of the magnetic carrier alone.

Microorganisms have been widely used as effective bioremediation materials in various fields of environmental remediation. The application of immobilization technology, in turn, provides a more stable living environment for microorganisms and enables microbial cells to maintain higher activity than free cells do in harsh environments. However, immobilization of microbial cells also has some disadvantages, such as the possibility of cell inactivation during immobilization, reduced microbial activity due to mass transfer limitations, accumulation of toxic metabolites in the carrier, and uncontrolled cell growth in the blocked region, leading to cell leakage [22]. Therefore, maintaining the activity and productivity of microbial cells in immobilized systems and enhancing the performance of carrier materials has become the main focus in the application of immobilized microbial technology.

**Table 2:** Application of immobilized microorganism in the treatment of refractory organics contaminants contained in waste water.

Microorganisms	Carrier	Immobilization Method	Contaminant	Ref.
*Halomonas* and *Aneurinibacillus*	Straw-alginate	Entrapment	Diesel	[41]
*Pseudomonas moorei KB4*	Loofah sponge	Adsorption	Paracetamol	[93]
*Seudomonas citronellolis*	Biochar	Adsorption	Biodegradation	[94]
Consortium GYB1	Alginate-biochar	Entrapment	2,3′,4,4′,5-pentachlorodiphenyl	[58]
*P. putida*	Biochar	Covalent binding-Adsorption	Paraquat	[95]
*P. putida*	AC	Adsorption	phenol	[96]
*Bacillus thuringiensis* B1	XAN-PDA	Cross-linking	Naproxen	[49]
*Saccharomyces pastorianus*	Alginate	Entrapment	Ethacridine lactate	[97]

#### 2.3.2. Enzymes

Enzymes are efficient natural catalysts with high activity, substrate specificity, and selectivity compared with traditional catalysts [98,99]; however, enzymes are soluble substances that are not conducive to separation and recovery. Residual enzymes may also cause pollution, and most of them are sensitive to the external environment and highly susceptible to inactivation [99]. Immobilization technology is an effective way to solve these problems. The immobilized enzyme can be easily separated from the reaction system so that the enzyme can be continuously produced, and it has been widely used in practical production. As early as 1916, Nelson and Griffin demonstrated that immobilized invertase on charcoal materials could maintain catalytic activity in aqueous environments [100].

Immobilized enzymes have great potential for environmental remediation (Table 3). Masjoudi et al. [101] immobilized laccase for the removal of the organic drug diclofenac and found that the operational stability of the immobilized enzyme was significantly enhanced, maintaining more than 20% of the initial activity after five repetitions, whereas the removal efficiency of the enzyme for diclofenac reached 95% within 4 h after immobilization. Immobilized horseradish peroxidase (HRP) has been shown to be effective in removing the carcinogen aflatoxin B1 (AFB1), and the immobilized enzyme maintained 65% removal after five cycles [102].

During enzyme immobilization, the rate of enzymatic reactions is usually described using the Michaelis–Menten equation (Equation (1)) [103]:

(1) $$v=\frac{v_{max}\left[S\right]}{k_{m}+\left[S\right]}\;$$

where *v* is the reaction rate, *k_m_* is Michaelis constant, *v_max_* is the reaction rate of the enzyme at substrate saturation, and [*S*] is the substrate concentration. 

It has been found that the increase in *k_m_* values and decrease in *v_max_* values after immobilization indicate a decrease in the affinity between the enzyme and the substrate and a decrease in the maximum reaction rate, which may be due to structural changes in laccase during immobilization and mass transfer limitations to the substrate and product molecules [104].

Overall, immobilization technology enhances the thermal stability of enzymes and their resistance to adverse environments [105] while the reaction conditions are milder [106], compensating for the disadvantages of free enzymes, such as poor storage, poor operational stability, and difficult recovery [107]. However, immobilized enzymes still have limitations; for example, immobilized carriers can affect mass transfer efficiency, and enzyme activity can be affected by the carrier, which affects the removal of pollutants. In addition, the use of enzymes as biodegradable materials in large-scale water treatment is limited by their high cost. In contrast, the use of low-cost microorganisms to treat wastewater is a trend in industrial applications [108].

**Table 3:** Application of immobilized enzyme in the treatment of refractory organics contaminants containedin waste water.

Enzyme	Carrier	Immobilization Method	Contaminant	Ref.
Polyphenol oxidase	Chitosan-montmorillonite	Adsorption	Phenolic compounds	[109]
Laccase from *Aspergillus oryzae*	Graphene Oxide	Adsorption	Malachite Green	[110]
Laccase from *Aspergillus oryzae*	Porous geopolymer	Cyclic adsorption	Crystal violet	[111]
Laccase from *Pycnoporus sanguineus* (CS43)	Multi-channel ceramic membrane	Covalent bonding	BPA	[112]
Soybean peroxidase	Fe_3_O_4_@SiO_2_ particles	Covalent bonding	Malachite green	[113]
Laccases from *T. pubescens*	Alginate-glutaraldehyde	Cross linking-Entrapment	BPA	[114]
Tyrosinase from *Penicillium chrysogenum*	Alginate	Entrapment	Phenol	[115]
Laccase	CoCu-MOF	Entrapment	Congo red	[116]

## 3. Removal of Refractory Organic Pollutants in Wastewater

Currently, with industrial development, the amount of wastewater discharged is increasing daily, and the removal of refractory organic compounds from wastewater has become one of the main concerns of the public. The removal of refractory organic pollutants such as phenol compounds, pesticides, medical wastewater, synthetic dyes, and surfactants is crucial because they are ubiquitous in the environment and pose a serious threat to ecosystems and human health [117]. Previous studies have found that immobilized biomass materials can be effectively used for the removal of refractory organic pollutants from wastewater (Table 4).

### 3.1. Removal of Organic Pollutants by Immobilized Microorganisms

Immobilized microorganisms have shown great potential for the removal of organic pollutants. Yu et al. [63] immobilized *Arthrobacter* sp. ZXY-2 on BC to remove the pesticide atrazine; in that study, a high removal percentage was obtained in a short time period: complete removal of 50 mg/L atrazine within 1 h. The degradation pathways include dealkylation, alkyl hydroxylation, dichlorination-hydroxylation, and alkyl oxidation. The addition of carrier BC enhanced the binding force between ZXY-2 and the pollutant and improved its atrazine degradation rate. Pongkua et al. [118] studied the immobilization of *Acinetobacter indicus* on sulfuric acid–modified bagasse-activated carbon-bone BC beads. Beads were utilized for the biodegradation of gaseous methyl tert-butyl ether (MTBE), and the results demonstrated that the prepared biocatalyst achieved 90% degradation of MTBE within 3 h with the attached growth of *Acinetobacter indicus*. After eight cycles, the biocatalyst could continue to degrade pollutants as new nutrient sources were added. Deng et al. [119] utilized modified peanut shell powder (PSP) to immobilize *Mycobacterium gilvum* and achieved 98% removal of 10 mg/L pyrene (PYR) within 7 days. The study demonstrated that the immobilized cells showed more significant advantages at higher PYR concentrations than free microorganisms did; this is likely because the immobilized carriers provided a good growth environment for the microorganisms to maintain a faster proliferation rate even at higher PYR concentrations. Partovinia et al. [120] investigated the degradation performance of immobilized microbial flora (obtained from the activated sludge of the Tehran refinery) on the polycyclic aromatic hydrocarbon phenanthrene (PHE). The results showed that the immobilized microorganisms achieved complete removal of 250 ppm PHE within five days and maintained effective removal of PHE after nine cycles, while immobilized microorganisms were found to have a better ability to remove hydroxylated PHE (IMs), an intermediate metabolite in the degradation process, than to remove free microorganisms. Huang et al. [121] utilized mixed flora to remove the organic pollutant benzo[a]pyrene (BaP) and noticed mutual repulsion between degrading flora; however, the technique was used to immobilize these degrading bacteria individually, which could effectively reduce the repulsion between degrading flora and improve the degradation efficiency of BaP.

Microorganisms immobilized on the carrier proliferate and grow, forming an extremely ecosystem-rich biofilm with good removal of refractory organic contaminant. Tang et al. [122] used a biofilm reactor to treat pharmaceutical-containing wastewater and obtained a more than 50% removal rate of diclofenac and atenolol. Tombola et al. [123] used recycled corrugated wire hose cover as carriers placed in a biofilm reactor for wastewater treatment. It was found that the reactor had a good removal effect on various refractory organics including naproxen and trimethoprim, and the removal rates of all pollutants were above 85%. Tian et al. [124] used a biofilm reactor for degrading phenolic compounds in high saline wastewater, and found that γ-proteobacteria played a major role in the biofilm, and more than 90 % of phenol degradation rate was maintained within 90 days at stable operation.

Previous studies have confirmed that immobilized microorganisms can be applied for the removal of a wide range of refractory organic pollutants and are mostly adaptable to complex external environments [94]. However, cell leakage can occur in immobilized systems because of diffusion effects. Therefore, research and application of immobilized microorganisms can focus on the effective combination of microorganisms with immobilized carriers and the mass transfer efficiency of pollutants in immobilized systems.

### 3.2. Removal of Organic Pollutants by Immobilized Enzyme

In recent years, research into the use of immobilized enzymes to degrade organic pollutants has received extensive attention [125,126]. Vineh et al. [127] used modified reduced graphene oxide (RGO) to covalently immobilize HRP. The results showed that the immobilized HRP had a removal efficiency of up to 100% for high-phenol-concentration wastewater (2500 mg/L), while the removal of phenol by free HRP was only 55%. Further investigations revealed that there was a synergistic effect between the immobilized HRP and RGO through covalent bonding, which enabled HRP to maintain high activity during biodegradation, thus achieving the desired removal effect. Petronijević et al. [45] prepared an immobilized BC-HRP material for phenol degradation by immobilizing HRP onto BC. In this study, a high degradation percentage was obtained in a short time period: a 90% degradation rate in 2 h. After five cycles, the removal efficiency of phenol was still 64%, and the immobilized HRP could still maintain 20% activity at 80 °C. Mechanistic studies demonstrated that the hydrophobic group on the carbon material enhanced the affinity of the enzyme for phenolic compounds, while immobilization resulted in a synergistic effect between the carbon carrier and HRP. In addition, immobilized redox enzymes are effective biocatalytic materials and have great potential for water treatment. Bilal et al. [128] achieved complete removal of BPA utilizing chitosan immobilized laccase, while the immobilized laccase exhibited a high degree of stability, with residual enzyme activity exceeding 90% even when stored at 4 °C for 28 days. Fan et al. [129] immobilized chloroperoxidase (CPO) on both the inner and outer walls of Halloysite nanotubes (HNT) to obtain the immobilized material I-CPO. In this study, I-CPO completely degraded 26.7 μmol/L of isoproturon in only 10 min, indicating that I-CPO has potential application in pesticide wastewater treatment. Wen et al. [40] utilized acid-base-modified bentonite-derived mesoporous materials (BDMMs) to immobilize laccase to prepare BDMMs-Lac for tetracycline (TC) removal. The results showed that BDMMs-Lac was able to remove 60% of TC within 120 min, and its thermal stability was greatly improved compared to that of free laccase. This study proved that BDMMs are novel, environmentally friendly, low-cost, reusable immobilized laccase carriers with potential applicability in the immobilization of biomolecules.

Enzyme immobilization technology is very effective for the removal of refractory organic compounds from wastewater, and its removal ability is related to both the immobilized carriers and enzymes, most of which have a synergistic effect. However, the presence of immobilized carriers makes it difficult for substrate molecules to interact with enzymes, causing spatial blockage of the active site of the enzyme, which leads to lower enzyme activity and a lower reaction rate. Therefore, for the research on and application of immobilized enzymes, the diffusion limitation problem in the reaction system can be overcome by developing excellent carriers.

**Table 4:** Parameters of immobilized enzyme for Organic pollutant removal.

Immobilized Biomass	Contaminant	Immobilization Method	Initial Concentration	Degradation Efficiency	Ref.
Biochar-*Bacillus cereus* LZ01	Chlortetracycline	Adsorption	75 mg/L	83%, 2 d	[130]
Pine needle biochar-Laccase	Malachite green	Adsorption	50 mg/L	85%, 5 h	[131]
*Bacillus subtilis*	Methylene blue	Covalent binding	100 mg/L	95%, 3 h	[132]
Fe_3_O_4_-*Penicillium sp. yz*11-22N2	Atrazine	Entrapment	8 mg/L	91.2%, 5 d	[133]
Bamboo charcoal-Microbial community	Nonylphenol	Adsorption	50 mg/L	69.5%, 8 d	[134]
Alginate-Laccase	BPA	Cross-linking	20 mg/L	99%, 2 h	[114]
Zeolite-Laccase	2,4-Dinitrophenol	Covalent binding	1.5 mg/L	100%, 6 h	[135]
Montmorillonite-Laccase				90%, 6 h	

## 4. Factors Affecting the Application of Immobilized Biomass Materials

The application of immobilized biomass materials is influenced by many factors, such as environmental conditions, operating temperature, pH, and biomass concentration.

### 4.1. Effect of Temperature

Temperature affects microorganism/enzyme activity and the mechanical properties of some carrier materials, which in turn affects the application of immobilized biomass materials in bioremediation. Ariaeenejad et al. [136] investigated the performance of GO immobilized on a model enzyme (PersiManXyn1) for the removal of methyl blue (MB) dye in water. The results showed that the removal efficiency of MB increased from 31% to 78% within 180 min as the temperature increased from 25 °C to 45 °C. In another study, researchers found that the removal of BPA using immobilized laccase increased continuously as the temperature was increased to 45 °C. However, experiments also revealed that a further increase in temperature led to a decrease in the removal rate [137]. These data coincided with the optimum temperature of the enzyme, indicating that the ability of the immobilized enzyme to remove contaminants is closely related to the operating conditions of the enzyme. In addition, the effect of temperature on the spatial structure and strength of the carrier is also evident, which in turn impacts the immobilization effect [19]. Flores et al. [138] used genipin as a cross-linking agent to immobilize enzymes on chitosan. The study found that the reaction between genipin and chitosan was slower at lower temperatures, creating a better spatial matrix for enzyme immobilization and thus achieving higher immobilization efficiency. Too high temperatures will accelerate molecular movement and promote the cross-linking reaction, but high temperatures are not favorable for enzyme immobilization because of the unstable nature of genipin and its ease of decomposition above 60 °C.

### 4.2. Effect of pH

pH is one of the most significant factors affecting immobilized biomass materials in practical applications and may affect the activity of the biomass and the functional groups on the surface of the material during the reaction process. During application, the activity of immobilized microorganisms and enzymes can be inhibited under strongly acidic and alkaline conditions, reducing the efficiency of pollutant removal. Zhu et al. [139] demonstrated that the removal of atrazine using immobilized *S. cerevisiae* increased continuously from 55% to 85% when the pH of the solution was increased from 3 to 7; however, the removal decreased to 60% when the pH was increased to 9. On the other hand, immobilization can also increase the tolerance of biomass materials to environmental pH. For example, Wang et al. [140] utilized magnetic shell-core MOFs to immobilize laccase for alkylphenol ethoxylate compound; the results showed that the optimum pH for both immobilized and free laccase was 5. However, the residual activity of immobilized laccase was 1.7 times higher than that of free laccase at a pH of 3, indicating that immobilized enzymes exhibited a wider pH tolerance range than free enzymes did. Furthermore, it has been shown that environmental pH affects the difference in surface charge and the interaction between the biomass materials and carrier, which in turn affects the performance of the immobilized biomass materials [48]. For example, Fan et al. [141] showed that the immobilized carrier SA/cellulose nanocrystal/PVA had a positive surface charge when the solution pH was <5.3 and a negative surface charge when the pH > 5.3, while at a pH > 4, the COOH in diclofenac sodium (DS) was ionized, mainly in the form of anions, so there was mutual repulsion between the carriers and the DS at a pH > 5.3, resulting in a sharp decrease in the DS removal capacity of the carriers. Therefore, choosing an appropriate pH is beneficial for enhancing the removal of pollutants by immobilized carrier materials.

### 4.3. Effect of Biomass Materials Concentration

When immobilized biomass materials are used in environmental remediation, the concentration of biomass materials often affects the performance of the material in removing contaminants. Within a certain range, microbial/enzyme activity increases with concentration, and when the optimal level is reached, higher concentrations reduce microbial/enzyme activity. On the one hand, microbial overgrowth can clog the pores on the surface of immobilized materials and reduce their mass transfer effect; on the other hand, this may be due to the interaction of adjacent microbial/enzyme molecules, resulting in partial loss of biomass activity. Zhang et al. [142] studied the removal of trichlorfon (TCF) by immobilizing *Aspergillus sydowii* (*A. sydowii*) on magnetically separable chitosan beads (MCBAs). Their results showed that for *A. sydowii* spore concentrations of 5.2 × 10^4^, 5.2 × 10^5^, and 5.2 × 10^6^ CFU/mL (CFU, Colony-Forming Units), the removal capacity of MCBAs for TCF was 80.38, 109.91, and 135.43 mg/g, respectively, indicating that relatively higher *A. sydowii* concentrations had higher removal rates for TCF. Another study also confirmed that the activity of the enzyme during the immobilization process can achieve efficient removal of pollutants within a certain range; however, when the enzyme concentration is too high, it will lead to a lack of inter-binding regions and adsorption sites between the carrier material and the enzyme, which hinders electron transfer and thus leads to a decrease in enzyme activity [143]. Therefore, to achieve the best performance of immobilized biomass materials, the biomass concentration needs to be carefully considered in the preparation of materials so that the concentration is not too high or too low, which reduces the activity of the biomass and thus affects the removal of pollutants.

## 5. Applications of Immobilized Biomass Materials in Bioreactor

Immobilized biomass materials in a reactor are effective in avoiding microbial/enzyme detachment and preserving the activity and performance stability of biomass through continuous operation. In addition, reactor design flexibility and operational stability are advantages of immobilized biomass materials in practical applications, and the type of reactor plays an important role in the development of the process [144].

**Figure 4:**
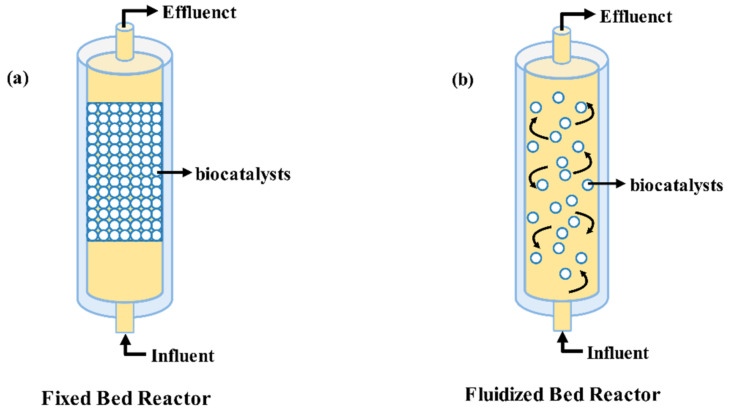
Schematic diagram of fixed bed reactor and fluidized bed reactor: (**a**) fixed bed reactor, (**b**) fluidized bed reactor.

Fixed-bed reactors (Figure 4a) are simple in design, easy to operate, and widely used in environmental management [87]. In recent years, research on the use of immobilized biomass materials as a filler in fixed-bed reactors to treat organic wastewater has gradually emerged, and its contribution to environmental sustainability has been significant. Mohanty et al. [145] utilized corn cob BC to immobilize microbial consortia from textile wastewater and used it as a filler in a continuous up-flow fixed-bed reactor for the removal of indanthrene blue RS. The results showed that the maximum adsorption capacity of RS was 4.55 mg/g under optimal conditions (pH of 10.0, temperature of 30 ℃, and an inoculum amount of 3.0 × 10^6^ CFU/mL). This study demonstrates that immobilized biomass materials as a filler in a reactor has been successfully used for the decolorization of dye wastewater on an industrial scale. In another study, Erhan et al. [146] utilized immobilized *Pseudomonas syringae* for phenol degradation in a fixed-bed column bioreactor. The study showed that the reactor achieved 100% degradation of phenol during continuous operation of the bioreactor when the phenol concentration was 200 mg/dm^3^ and the flow rate was less than 10 cm^3^/min. Xia et al. [147] immobilized laccase on polyethyleneimine-functionalized magnetic nanoparticles, which were filled with magnetic laccase in a novel fixed-bed bioreactor. The study demonstrated that, after continuous degradation of phenolic compounds for 18 h, the degradation rate in the reactor was 2.38 times that of the batch treatment, and, under optimal operating conditions, the fixed-bed reactor still maintained a phenol degradation rate of more than 70% after continuous operation for 48 h.

The fluidized bed reactor (Figure 4b) has better heat and mass transfer performance than the fixed-bed reactor does, which can maintain the immobilized beads in suspension, thus exhibiting a higher pollutant removal effect. Ferreira et al. [148] immobilized *Pseudomonas stutzeri* CECT 930 on agar as a biofiller in a fluidized bed reactor to remove groundwater PHE. When the initial concentration of PHE in the system was 100 μmol/L, the reactor quickly reached its steady state and achieved 96% removal. Wang et al. [149] utilized magnetic mesoporous silica nanoparticles to immobilize laccase in a fluidized bed reactor to achieve efficient degradation of phenol in coking wastewater. When the flow rate was less than 45 mL/h, the system achieved more than 99% degradation of phenol wastewater with an initial concentration of 100 mg/L in the continuous reactor, which was higher than the 69.2% degradation for indirect treatment, and the degradation rate remained above 90% after 40 h of continuous operation. Lassouane et al. [150] immobilized laccase using cross-linking–entrapment technology as a filler in a fluidized bed reactor for the degradation of BPA. The results of the study showed that the removal rates of BPA in the reactor were 98.4% and 96.5% at BPA concentrations of 60 mg/L and 80 mg/L, respectively. When the concentration was increased to 100 mg/L, the biodegradation rate of BPA remained above 75%, and the performance of the system was quite stable. In actual production, the fluidized bed reactor with immobilized biomass materials as a filler showed excellent reaction activity and system stability, which provided an effective method for the continuous removal of organic pollutants from industrial wastewater.

Biofilm reactors have been extensively studied in the field of treating refractory organic contaminants. Spennati et al. [151] found that a fixed bed biofilm reactor inoculated with *Aspergillus tubingensis* MUT 990 have a destructive effect on the removal of tannins. Kumar et al. [152] prepared a fluidized bed biofilm reactor for the removal of microcystin-LR from wastewater, which used *A. ramosus* and *Bacillus sp.* as degrading bacteria, respectively. They found that the reactor achieved 93% and 90% degradation rates for these two different microbes, and after 1.5 days of reaction, this reaction treated 200 m^3^ wastewater with high efficiency and economy.

At present, research on and application of immobilized biomass materials as a filler in reactors to remove organic wastewater are still rare, and many problems occur in existing application cases; for example, the residence time of pollutants in bioreactors is too short to achieve effective contact with immobilized biomass materials at higher flow rates, resulting in low pollutant removal rates. However, as a novel and efficient environmental functional material, the application of immobilized biomass materials to reactors to treat polluted environments has favorable results. Therefore, research in this area needs to be strengthened further.

## 6. Conclusions

This research demonstrates that immobilized biomass materials have higher stability, better resistance to harsh environments, and better recovery and reusability than free biomass does, making this technology a promising research direction. This review focuses on biomass materials immobilization in organic wastewater treatment in recent years and discusses the roles of immobilization methods, carriers, and biomass materials in immobilization technology; the significance of biomass materials immobilized in environmental bioremediation was affirmed. 

## 7. Perspectives

Although immobilization technology is rapidly developing, it faces various challenges that need to be further studied:

(1) The development of novel efficient and inexpensive immobilization carriers is crucial. At present, immobilization technology has been widely studied in the field of wastewater treatment; however, industrial-scale applications are limited by the composition of wastewater, operating conditions, and other factors. In particular, the price and service life of carrier materials are key factors for the economic feasibility of immobilization technology. Appropriate carriers and corresponding immobilization methods are the basis for success; therefore, seeking carriers with low cost, high stability, and excellent biocompatibility may become a new topic in this field.
(2) Maintenance of biological activity and mass transfer efficiency during immobilization is the core technology of immobilization methods. Although biomass materials immobilization is economical, efficient, recyclable, and adaptable to environmental changes, most immobilization methods lead to biomass materials deactivation, and traditional immobilization techniques often affect the mass transfer efficiency between the biomass materials and substrate. Therefore, it is crucial to develop better immobilization methods to address the shortcomings of traditional methods for more efficient engineering applications.
(3) If a single immobilization method cannot effectively achieve biomass immobilization, two or more immobilization methods can be combined to enhance the immobilization process; for example, using the adsorption–entrapment method can simultaneously solve the low affinity of adsorption and the high mass transfer resistance of entrapment. Therefore, the choice of two methods that can complement each other for the composite immobilization of biomass materials can lead to better overall performance of the immobilized biomass materials.

## Data Availability

All data generated or analyzed during this study are included in this article. Further enquiries can be directed to the corresponding author.
